# An evolutionary game-based simulation study of a multi-agent governance system for smart senior care services in China

**DOI:** 10.1186/s12877-023-04521-w

**Published:** 2023-12-19

**Authors:** Qiannan Shi, Shumian Yang, Na Wang, Shu-e Zhang, Yanping Wang, Bing Wu, Xinyuan Lu, Yining She, Zhihao Yue, Lei Gao, Zhong Zhang

**Affiliations:** 1 School of Health Management, Harbin Medical University, No. 157 Baojian Road, Nangang District, Harbin, 150086 Heilongjiang China; 2 Medical Department, Heilongjiang Provincial Hospital, Harbin, Heilongjiang, China

**Keywords:** Smart senior care, Multi-agent governance, Evolutionary game, Stability strategy

## Abstract

**Background:**

The competing interests of the government, smart senior care technology service providers, and older adults have led to a serious fragmentation of governance in China. This study aims to identify the collaboration mechanisms and evolutionary stabilization strategies for these agents.

**Methods:**

An evolutionary game model is developed to analyze the strategic decisions made by the government, smart senior care technology service providers, and older adults. A sensitivity analysis is conducted using data from Anhui Province, China, to verify the effects of relevant parameters on the strategy decisions of each agent.

**Results:**

The results of the simulation and sensitivity analysis indicated that, first, despite changes in the initial willingness values of the tripartite agents, the system eventually converges on 1. Second, the collaboration mechanism of the tripartite agents in the smart senior care system is related to government incentives, penalties, and subsidies, smart senior care service costs, and the additional benefits provided to smart senior care technology service providers.

**Conclusion:**

The strategy decisions of the government, providers, and older adults interact with each other. To promote collaboration among the tripartite agents and improve governance effectiveness, the government should strengthen the regulations for providers, increase penalties for providers that engage in a breach of trust, provide moderate incentives and subsidies, and control smart senior care service costs.

## Introduction

Population aging has emerged as a significant trend of social development at the global scale in recent years [1–3]. In China, this trend is expected to persist [4] and have a considerable impact on the national economy and social services, including medical care. As a consequence of such impact and owing to the triple effect of the Internet, information, and sensing technologies, smart senior care has emerged as an optimal solution to deal with the aging population [2, 5, 6]. Smart senior care involves constructing an information exchange platform using internet, communication, and sensing technologies to create a livable living environment for older adults, thereby enhancing their satisfaction and quality of life [7].

To promote the development of the smart senior care industry in China, the Ministry of Industry and Information Technology, the Ministry of Civil Affairs, and the National Health and Welfare Commission have jointly formulated the *Action Plan for the Development of the Smart Senior Industry (2021–2025)* [8]. This action plan follows the principles of “government-led, and multi-agent linkage,” continuous strengthening of information technology support, and improvement of the senior care service industry capacity [8]. In China, the “Internet + Aging” national policy has provided a technical solution to address the challenges posed by the country’s aging population [2]. Multi-agent governance forces have responded to this national policy by actively integrating it into the smart senior care model, and in order to improve older adult quality of life and autonomy. However, it remains a challenge to investigate multi-agent governance for smart senior care in China. From a horizontal perspective, the diversity of supplying agents entails divergent preferences and interests, which may lead to disputes and unbalanced interests. Additionally, the lack of clarity about agents’ rights and responsibilities coupled with governmental overstepping and misalignment may result in weak and uncoordinated governance, leading to a dilemma of fragmented governance. Thus, identifying the collaboration mechanisms and balancing the interests of various governance agents in the context of such services is crucial. We need to find strategies that equalise the interests of the tripartite governance agents, i.e. stability strategies. These descriptions show the need for multi-agent governance collaboration strategies that support the current development mode of smart senior care in China [9, 10].

Coordinating the link between different agents is especially crucial because smart senior care development in China is still in its early and exploratory stages, and there is a fragmented governance challenge. This study's analytical framework is based on the welfare pluralism theory. The two core ideas of the welfare state—pluralism and decentralization—were initially articulated by the British academic Ross in 1986. He maintained that the three sectors of the state, the market, and the family work together to create social welfare rather than merely the government's role alone. This approach is referred to as the "welfare pluralist triad," or Ross's welfare pluralism. Its main goal is to encourage the involvement of various sectors in order to create a positive feedback loop for welfare services [11–14]. The cooperation between the government and smart senior care technology service providers, which focuses on the needs of the older adults, enhances their quality of life, and realizes multi-agents government of senior care services, is therefore the embodiment of welfare pluralism in the "smart senior care" model.

### Literature review

#### Research on the development of smart senior care services

Big data platforms, smart technologies, artificial intelligence, and the Internet of things have permeated every facet of smart senior care, bringing radical changes to the industry and influencing its sustainable development. Accordingly, some of the governance issues raised by these developments should be viewed rationally and further examined, and some researchers have actually investigated related problems. For instance, Hu [15] conducted a case study and showed the development trends and application status of the smart senior care model in rural China; the cited author suggests improvements for the related systems and policies through further research on the current status of, and practical barriers to, the application of smart senior care in the country. Goharinezhad [16] conducted semi-structured interviews with senior caregivers and provided policy recommendations on the current status of aging in Iran.

Regarding smart senior care service quality testing, Xu [17] built a Fuzzy-QFD model and performed a grey correlation analysis to examine the relationship between the needs of older adults and platform functions. She then proposed several quality improvement measures for smart senior care service platforms. Then, a case study [18] was conducted in Jiangsu Province to evaluate the study by Xu [17] and its quality assessment of smart community senior care services; the cited case study used a two-stage decision model and a grey comprehensive evaluation, conducted a thorough evaluation of the quality of smart community senior care services, and provided related decision-making suggestions. Moreover, Chen [19] used the Delphi method and hierarchical analysis to develop a smart home care quality evaluation index system, which was then evaluated and validated.

Regarding older adult privacy and security, Pirzad [20] examined older adults’ low utilization of smart home devices, concluded that smart homes have an impact on their lifestyle and privacy, and offered appropriate improvement methods. Schomakers [21] utilized a questionnaire to examine the similarities and differences in smart technology application and the effect of related privacy and security concerns on people’s attitudes and views of smart technologies. Therefore, these difficulties demonstrate that the current governance system of smart senior care is not yet flawless, whether it is Chinese study on the quality assessment of smart senior care or international research on the privacy and security of older adults. An in-depth analysis of the current governance issues surrounding smart senior care in China is required in order to support the advancement of the governance system and, at the same time, this study considers the level of understanding of the nation's national conditions and realities.

#### Research on multi-agent governance

To solve the aforementioned governance problems of smart senior care, research shows that we should consider multi-agent governance. Specifically, scientists have suggested several ways to improve cooperation among agents, and shown that diversified co-governance is developing as a significant trend for tackling social problems.

Regarding definition, the term multi-agent governance refers to cooperation between the public sector and other stakeholders in a mutually inclusive, transparent, and equitable environment to address social problems, including complex policy reforms [22, 23]. To enhance integrated care and achieve better results in health and social care services, Gordon [9] suggested enhancing collaboration between patients, service providers, policy researchers, and researchers, as well as that patient collaboration should be particularly emphasized. Sharma [24] strived to establish a cooperative framework for the neurosurgeons, the government, critical care unit specialists, nursing staff, and other stakeholders to be able to collaborate in improving access to and quality of neurosurgical services. In her exploration of the difficulties associated with knowledge translation in the three fields of academia, industry, and medicine, Li [25] makes the case for the need to improve and create specific policies for triangular cooperation and communication.

Through interviews and case studies, Ehrenhard [26] created a general value network model for smart home technologies to identify market obstacles, and that can yield suggestions for appropriate solutions to boost the adoption of such technologies from the perspective of each stakeholder. Schiavone [27] examined the impact and challenges of digital business models and shared mobility in the healthcare ecosystem from a multi-stakeholder perspective. Podgórniak-Krzykacz [28] investigated the viability of establishing smart cities and senior communities in Poland in the context of the country’s aging population; the author suggested the following measures to further promote these concepts in the country: cross-sectoral cooperation, social participation promotion, and government policy support. Using a thorough interdisciplinary approach, Fiona et al. [29] analyzed the benefits and limitations of pharmacy interventions to increase vaccination rates and offered useful suggestions for improving public health. In summary, and as aforementioned, multi-agent governance has become a new perspective for solving social governance problems. It is noteworthy that while international research on multi-agent governance offers significant insights and theoretical underpinnings, research on the multi-agent governance of smart senior care in China is still necessary due to the country's different institutional mechanisms and regimes. This is necessary in order to develop strategies and policies that are appropriate for China's institutions and regimes and to advance the development of multi-agent governance governance of smart senior care.

#### Research on evolutionary game theory application

Macro-policy research [30] and qualitative analyses [5, 31, 32] make up most of the literature currently available on smart senior care. Meanwhile, related quantitative [33, 34] research have typically used static designs focused on optimization. Thus, despite governance being a dynamic process, few studies have used systematic and dynamic designs to explore the influence and trends of each governance agent’s strategy from a micro perspective.

Evolutionary game theory exploits dynamic games and agents’ finite rationality. It combines evolutionary theory and game theory, and is used to describe how people behave in cooperative games. Nash equilibrium occurs when all agents’ strategies are in sync and there is no motivation to modify them, whereas evolutionary stable strategies cannot be easily replaced during system evolution [35, 36]. In contrast to traditional game theory, evolutionary game theory assumes that agents are imperfectly rational and their strategies are dynamically adjusted [37]. Moreover, agents can still reach a Nash equilibrium even if they lack all the information by learning about the benefits and drawbacks of strategic decision changes [38]. This theory has been extensively applied in many fields, such as public administration [39, 40], environmental regulation [41], new energy [42], coal mine safety regulation [43], healthcare [44, 45], and epidemic transmission and traffic flow [46]. Additionally, there are numerous parameters that affect the game agents' strategic decisions, making it unlikely that equilibrium may be reached quickly. Agents must instead gradually approach equilibrium by continually modeling, learning, and refining their own strategies. The agent's Nash equilibrium state in this case is the agent's stability strategy. Thus, evolutionary game theory and related models have already been proven to be useful methods for resolving social issues.

The conflicts of interests of various agents in smart senior care are consistent with the features of the evolutionary game. Thus, the current study used an evolutionary game model to assess each governance agent’s stability strategy in the Chinese environment; a sensitivity analysis was performed to investigate the important factors influencing strategy decisions. We hope that this study yields relevant data that supports the creation of collaboration mechanisms for smart senior care in China, which may in turn help address the problem of governance fragmentation, improve governance effectiveness, and balance the interests of various governance agents.

## Methods

### Basic assumptions

By analyzing the competing interests of the government, smart senior care technology service providers, and older adults, this study assesses the conundrum of the tripartite game system of smart senior care services. To promote and ensure the development and high-quality of smart senior care services, the government—as the head of the system—must financially subsidize both older adults and smart senior care technology service providers, as well as regulate the behavior of providers. These actions count as governmental strategies, and they not only emphasize the government’s leadership role but also provide the government with intangible advantages, such as appreciation from the public.

The advent of smart senior care technology service providers, which focus on addressing the various needs of older adults and serve as representatives of market forces, has significantly decreased the burden of the aging population on the Chinese government. The main interest of these providers is increasing own economic revenue and brand influence.

**Fig. 1:**
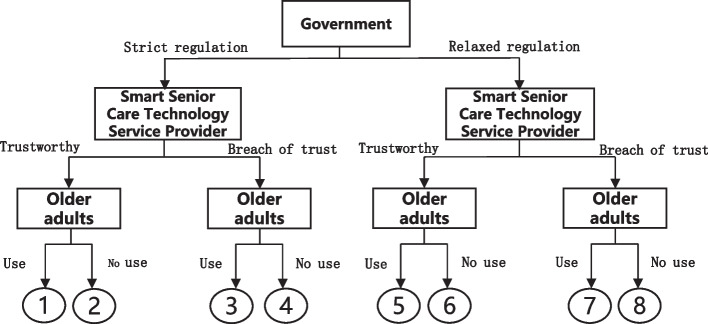
Tree decision of the tripartite evolutionary game agents in this study

**Fig. 2:**
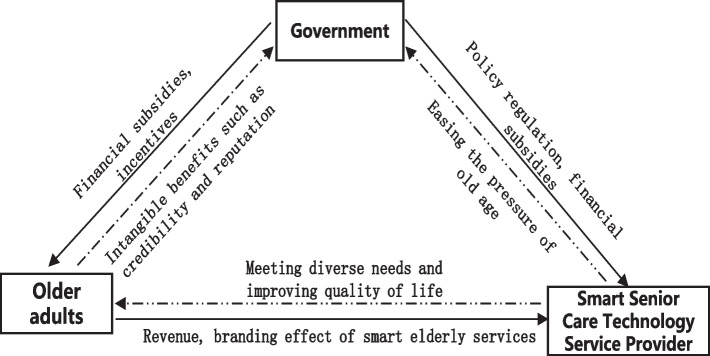
Logic diagram of the tripartite evolutionary game agents in this study

Older adults are the main users of the aforementioned smart senior care services, using them to satisfy their various needs, raise quality of life, and enjoy own spirituality, and they worry about topics such as costs and service quality. As illustrated in Figs. 1 and 2, this study created a tree decision and logic diagram, respectively, of the tripartite evolutionary game agents to allow for a more intuitive description of agent interactions. Below we present the study hypotheses.

Hypothesis 1: the combination of the tripartite game agents can be represented as $Q$, $Q = \left\{ {g, \, c, \, e \, } \right\}$. In this model, the set of $g$ symbolizes the government, $c$ symbolizes smart senior care technology service providers, and $e$ symbolizes older adults. All three are finite rational decision-makers because there is knowledge asymmetry between them.

Hypothesis 2: $Pg \,$ = {strict regulation, relaxed regulation} describes the combination of strategies of the government. Specifically, “strict regulation” refers to the government’s strict adherence to the issued directives and guidelines aimed at identifying and exposing service providers who break the law, fall short of industry standards, or jeopardize the rights and interests of older adults. “Relaxed regulation” refers to the government’s passive attitude toward smart senior care technology service providers’ betrayal of older adults’ confidence and legal violations.

Moreover,$Pc$ = {trustworthy, breach of trust} describes the combination of strategies of smart senior care technology service providers. “Trustworthy” refers to the providers’ actions toward guaranteeing service quality and preserving older adults’ legal rights and interests. A “breach of trust” occurs when a provider offers subpar services to older adults to increase own profits.

Furthermore, $Pe$ = {use, no use} describes the combination of strategies of older adults. In particular, “use” refers to older adults and their families making a conscious decision to use smart senior care services to enhance their quality of life. “No use” refers to older adults and their families adopting a reserved attitude toward services, not accepting them, or accepting them in an non-engaging manner.

Hypothesis 3: the probability of the government choosing “strict regulation” is $x$(0 ≤ $x$ ≤ 1), and that of choosing “relaxed regulation” is 1-$x$. The probability of a provider choosing “trustworthy” is $y$(0 ≤ $y$ ≤ 1), and that of choosing “breach of trust” is 1-$y$. Moreover, the probability of older adults choosing “use” is $z$(0 ≤ $z$ ≤ 1), and that of choosing “no use” is 1-$z$.

The relevant model parameters and meaning of parameters for the tripartite game agents are listed in Table 1.

**Table 1:** Model parameters and implications

Agents of the game	Model parameters	Meaning of parameters
Government	$M_{g1}$	The cost of strict government regulation
	$M_{g2}$	The cost of relaxed government regulation $\left( {{M_{g1}} > {M_{g2}}} \right)$
	$S_{g1}$	Benefits of strict government regulation (e.g., reputation and incremental performance)
	$S_{g2}$	Some reputational damage, loss of governance, and loss of trust when the government relaxes regulations of smart senior care technology service providers
Smart senior care technology service provider	$M_{c1}$	The cost of being trustworthy for smart senior care technology providers
	$M_{c2}$	The cost of a breach of trust for smart senior care technology providers $\left( {{M_{c1}} > {M_{c2}}} \right)$
	$N_c$	Government strictly regulates rewards for the trustworthiness of smart senior care technology providers
	$B_c$	Government provides subsidies for smart senior care technology service providers
	$Sc$	The brand effect and reputation gained by the trustworthiness of the smart senior care technology service provider when older adults use its services
	$c$	Additional benefits received by smart senior care technology service providers in the event of a breach of trust
	$L_{c1}$	Strict government regulation imposes fines on smart senior care technology service providers for a breach of trust
	$L_{c2}$	Smart senior care technology providers compensate older adults for a breach of trust
Older adults	$M_e$	The purchase cost of smart senior care services for older adults
	$H_{e1}$	Older adults’ use of smart senior care services to meet their diverse needs, improve quality of life and satisfaction, and for other benefits when the smart senior care technology provider is trustworthy
	$H_{e2}$	Older adults’ use of smart senior care services to meet their diverse needs, improve quality of life and satisfaction, and for other benefits when the smart senior care technology service provider breaches their trust $\left( {{H_{e1}} > {H_{e2}}} \right)$
	$N_e$	Government rewards for older adults who actively use smart senior care services when they are strictly regulated
	$B_e$	Government subsidies for older adults using smart senior care services

### Model construction

The payment benefit matrix for the tripartite game agents—the government, the provider of smart senior care technology services, and older adults—is displayed in Table 2 based on the foregoing hypothesis.

**Table 2:** Tripartite evolutionary game payment matrix

Agents of the game		Government	
Older adults	Smart Senior Care Technology Service Provider	Strict regulation **(**$x$**)**	Relaxed regulation **(**$1 - x$**)**
Use**(**$z$**)**	Trustworthy**(**$y$**)**	$$\begin{gathered} \begin{array}{*{20}{l}} {{S_{g1}} - {M_{g1}} - {B_c} - {B_e} - {N_c} - {N_e},} \\ {{N_c} + {B_c} + {S_c} + {M_e} - {M_{c1}},} \end{array} \hfill \\ {N_e} + {B_e} + {H_{e1}} - {M_e} \hfill \\ \end{gathered}$$	$$\begin{gathered} \begin{array}{*{20}{l}} { - {M_{g2}} - {B_c} - {B_e},} \\ {{B_c} + {M_e} + {S_c} - {M_{c1}},} \end{array} \hfill \\ {B_e} + {H_{e1}} - {M_e} \hfill \\ \end{gathered}$$
	Breach of trust **(**$1 - y$**)**	$$\begin{gathered} \begin{array}{*{20}{l}} {{S_{g1}} + {L_{c1}} - {M_{g1}} - {B_c} - {B_e} - {N_e},} \\ {{B_c} + c + {M_e} - {L_{c1}} - {L_{c2}} - {M_{c2}},} \end{array} \hfill \\ {L_{c2}} + {N_e} + {B_e} + {H_{e2}} - {M_e} \hfill \\ \end{gathered}$$	$$\begin{gathered} \begin{array}{*{20}{l}} { - {M_{g2}} - {B_c} - {B_e} - {S_{g2}},} \\ {{B_c} + c + {M_e} - {M_{c2}} - {L_{c2}},} \end{array} \hfill \\ {L_{c2}} + {B_e} + {H_{e2}} - {M_e} \hfill \\ \end{gathered}$$
No use**(**$1 - z$**)**	Trustworthy**(**$y$**)**	$$\begin{gathered} \begin{array}{*{20}{l}} {{S_{g1}} - {B_c} - {N_c} - {M_{g1}},} \\ {{N_c} + {B_c} - {M_{c1}},} \end{array} \hfill \\ 0 \hfill \\ \end{gathered}$$	$$\begin{gathered} \begin{array}{*{20}{l}} { - {M_{g2}} - {B_c},} \\ {{B_c} - {M_{c1}},} \end{array} \hfill \\ 0 \hfill \\ \end{gathered}$$
	Breach of trust **(**$1 - y$**)**	$$\begin{gathered} \begin{array}{*{20}{l}} {{S_{g1}} + {L_{c1}} - {M_{g1}} - {B_c},} \\ {{B_c} + c - {L_{c1}} - {M_{c2}},} \end{array} \hfill \\ 0 \hfill \\ \end{gathered}$$	$$\begin{gathered} \begin{array}{*{20}{l}} { - {M_{g2}} - {B_c} - {S_{g2}},} \\ {{B_c} + c - {M_{c2}},} \end{array} \hfill \\ 0 \hfill \\ \end{gathered}$$

### Model derivation

The expected benefit $G_1$, $G_2$ for “strict regulation” and “relaxed regulation” by the government, and the average expected benefit $\overline {G_1}$ by the government are:

1 $$\begin{gathered} {G_1} = zy({S_{g1}} - {M_{g1}} - {B_c} - {B_e} - {N_c} - {N_e}) + z\left( {1 - y} \right)({S_{g1}} + {L_{c1}} - {M_{g1}} - {B_c} - {B_e} - {N_e}) \hfill \\ + \left( {1 - z} \right)y({S_{g1}} - {B_c} - {N_c} - {M_{g1}}) + \left( {1 - z} \right)\left( {1 - y} \right)({S_{g1}} + {L_{c1}} - {M_{g1}} - {B_c}) \hfill \\ \end{gathered}$$

2 $$\begin{gathered} {G_2} = zy( - {M_{g2}} - {B_c} - {B_e}) + z\left( {1 - y} \right)( - {M_{g2}} - {B_c} - {B_e} - {S_{g2}}) + \left( {1 - z} \right)y( - {M_{g2}} - {B_c}) \hfill \\ + \left( {1 - z} \right)\left( {1 - y} \right)( - {M_{g2}} - {B_c} - {S_{g2}}) \hfill \\ \end{gathered}$$

3 $$\overline {G_1} = xG1 + (1 - x) G2$$

It can therefore be concluded from Eqs. (1), (2) and (3) that the government's replication dynamics equation Eq. (4) is:

4 $$F(x) = \frac{dx}{{dt}} = x\left( {{G_1} - \overline {G_1} } \right) = x\left( {1 - x} \right)\left[ \begin{gathered} {S_{g1}} + \left( {1 - y} \right){L_{c1}} - {M_{g1}} + {M_{g2}} + \left( {1 - y} \right){S_{g2}} - z{N_e} \hfill \\ - y{N_c} \hfill \\ \end{gathered} \right]$$

The expected benefit $C_1$, $C_2$ for the “trustworthy” and “breach of trust” strategies by the smart senior care technology service providers, and the average expected benefit $\overline {C_1}$ are:

5 $$\begin{gathered} {C_1} = zx({N_c} + {B_c} + {S_c} + {M_e} - {M_{c1}}) + z\left( {1 - x} \right)({B_c} + {M_e} + {S_c} - {M_{c1}}) + x\left( {1 - z} \right)({N_c} + \hfill \\ {B_c} - {M_{c1}}) + \left( {1 - x} \right)\left( {1 - z} \right)({B_c} - {M_{c1}}) \hfill \\ \end{gathered}$$

6 $$\begin{gathered} {C_2} = zx({B_c} + c + {M_e} - {L_{c1}} - {L_{c2}} - {M_{c2}}) + z\left( {1 - x} \right)({B_c} + c + {M_e} - {M_{c2}} - {L_{c2}}) + x \hfill \\ \left( {1 - z} \right)({B_c} + c - {L_{c1}} - {M_{c2}}) + \left( {1 - x} \right)\left( {1 - z} \right)({B_c} + c - {M_{c2}}) \hfill \\ \end{gathered}$$

7 $$\overline {C_1} = y{C_1} + \left( {1 - y} \right){C_2}$$

The replication dynamic equation for the smart senior care technology service provider from Eqs. (5), (6) and (7) can therefore be derived Eq. (8) as:

8 $$F(y) = \frac{dy}{{dt}} = y\left( {{C_1} - \overline {C_1} } \right) = y\left( {1 - y} \right)\left[ {z\left( {{S_c} + {L_{c2}}} \right) + x\left( {{N_c} + {L_{c1}}} \right) - {M_{c1}} - c + {M_{c2}}} \right]$$

The expected benefit $E_1$, $E_2$ for the “use” and “no use” by the elderly, and the average expected benefit $\overline {E_1}$ by older adults are:

9 $$\begin{gathered} {E_1} = xy({N_e} + {B_e} + {H_{e1}} - {M_e}) + y\left( {1 - x} \right)({B_e} + {H_{e1}} - {M_e}) + x\left( {1 - y} \right)({L_{c2}} + {N_e} + {B_e} \hfill \\ + {H_{e2}} - {M_e}) + \left( {1 - x} \right)\left( {1 - y} \right)({L_{c2}} + {B_e} + {H_{e2}} - {M_e}) \hfill \\ \end{gathered}$$

10 $${E_2} = 0$$

11 $$\overline {E_1} = z{E_1} + \left( {1 - z} \right){E_2}$$

The replication dynamics equation for older adults from Eqs. (9), (10) and (11) can therefore be derived Eq. (12) as:

12 $$F(z) = \frac{dz}{{dt}} = z\left( {{E_1} - \overline {E_1} } \right) = z\left( {1 - z} \right)\left[ {y{H_{e1}} + x{N_e} + \left( {1 - y} \right){L_{c2}} + {B_e} - {M_e} + \left( {1 - y} \right){H_{e2}}} \right]$$

### Stability analysis

When Eqs. (4), (8) and (12) satisfy the condition of $F\left( x \right) = 0$, $[F\left( y \right) = 0$ and $F\left( z \right) = 0$, eight local equilibrium points for the pure strategy ($x$, $y$, $z$) and local equilibrium points for the mixed strategy ($x^{\ast}$, $y^{\ast}$,$z^{\ast}$) are obtained. Although there are many possibilities for a mixed-strategy equilibrium point in asymmetric evolutionary games, each agent often chooses a deterministic approach based on the information that one already possesses. This implies that the equilibrium point in an asymmetric evolutionary game must be a rigorous Nash equilibrium, which is purely a strategy equilibrium [47, 48]. Therefore, the system produces eight local equilibrium points, as follows: (0, 0, 0), (0, 0, 1), (0, 1, 1), (0, 1, 0), (1, 0, 0), (1, 0, 1), (1, 1, 0), (1, 1, 0), and (1, 1, 1).

The Lyapunov First Method [49] is used to determine equilibrium point stability. In this method, if all the eigenvalues of the Jacobi matrices are negative, the local equilibrium point is an evolutionary stable equilibrium point. If there are eigenvalues of zero and all other eigenvalues are negative, the stability of the local equilibrium point cannot be determined by the eigenvalues. If there are positive eigenvalues, the local equilibrium point is unstable. The determination of each equilibrium point is presented in Table 3.

13 $$\begin{gathered} J = \left[ {\begin{array}{*{20}{c}} {\frac{\partial F(x)}{{\partial x}}}&{\frac{\partial F(x)}{{\partial y}}}&{\frac{\partial F(x)}{{\partial z}}} \\ {\frac{\partial F(y)}{{\partial x}}}&{\frac{\partial F(y)}{{\partial y}}}&{\frac{\partial F(y)}{{\partial z}}} \\ {\frac{\partial F(z)}{{\partial x}}}&{\frac{\partial F(z)}{{\partial y}}}&{\frac{\partial F(z)}{{\partial z}}} \end{array}} \right] \hfill \\ \hfill \\ \end{gathered}$$

$$\left[ {\begin{array}{*{20}{c}} {(1 - 2x)\left[ {{S_{g1}} + \left( {1 - y} \right){L_{c1}} - {M_{g1}} + {M_{g2}} + \left( {1 - y} \right){S_{g2}} - z{N_e} - y{N_c}} \right]}&{x\left( {1 - x} \right) \, \left[ { - \left( {{L_{c1}} + {S_{g2}} + {N_c}} \right)} \right]}&{x\left( {1 - x} \right)\left( { - {N_e}} \right)} \\ {y\left( {1 - y} \right)\left( {{N_c} + {L_{c1}}} \right)}&{\left( {1 - 2y} \right)\left[ {z\left( {{S_c} + {L_{c2}}} \right) + x\left( {{N_c} + {L_{c1}}} \right) - {M_{c1}} - c + {M_{c2}}} \right]}&{y\left( {1 - y} \right)\left( {{S_c} + {L_{c2}}} \right)} \\ {z\left( {1 - z} \right){N_e}}&{z\left( {1 - z} \right)\left( {{H_{e1}} - {H_{e2}} - {L_{c2}}} \right)}&{\left( {1 - 2z} \right)\left[ {y{H_{e1}} + x{N_e} + \left( {1 - y} \right){L_{c2}} + {B_e} - {M_e} + \left( {1 - y} \right){H_{e2}}} \right]} \end{array}} \right]$$

**Table 3:** Equilibrium point determination status

Equalization points	Eigenvalue		
	$\lambda$ 1	$\lambda$ 2	$\lambda$ 3
1 (0, 0, 0)	${S_{g1}} + {L_{c1}} - {M_{g1}} + {M_{g2}} + {S_{g2}}$	${M_{c2}} - c - {M_{c1}}$	${L_{c2}} + {B_e} - {M_e} + {H_{e2}}$
2 (0, 0, 1)	${S_{g1}} + {L_{c1}} - {M_{g1}} + {M_{g2}} + {S_{g2}} - {N_e}$	${S_c} + {L_{c2}} - {M_{c1}} - c + {M_{c2}}$	${M_e} - {L_{c2}} - {B_e} - {H_{e2}}$
3 (0, 1, 1)	${S_{g1}} - {N_c} - {M_{g1}} + {M_{g2}} - {N_e}$	${M_{c1}} + c - {M_{c2}} - {S_c} - {L_{c2}}$	${M_e} - {H_{e1}} - {B_e}$
4 (0, 1, 0)	${S_{g1}} - {N_c} - {M_{g1}} + {M_{g2}}$	${M_{c1}} + c - {M_{c2}}$	${H_{e1}} + {B_e} - {M_e}$
5 (1, 0, 0)	${M_{g1}} - {S_{g1}} - {L_{c1}} - {M_{g2}} - {S_{g2}}$	${N_c} + {L_{c1}} - {M_{c1}} - c + {M_{c2}}$	${N_e} + {L_{c2}} + {B_e} - {M_e} + {H_{e2}}$
6 (1, 0, 1)	${M_{g1}} + {N_e} - {S_{g1}} - {L_{c1}} - {M_{g2}} - {S_{g2}}$	${S_c} + {L_{c2}} + {N_c} + {L_{c1}} - {M_{c1}} - c + {M_{c2}}$	${M_e} - {N_e} - {L_{c2}} - {B_e} - {H_{e2}}$
7 (1, 1, 0)	${N_c} + {M_{g1}} - {S_{g1}} - {M_{g2}}$	${M_{c1}} + c - {N_c} - {L_{c1}} - {M_{c2}}$	${H_{e1}} + {N_e} + {B_e} - {M_e}$
8 (1, 1, 1)	${N_c} + {M_{g1}} + {N_e} - {S_{g1}} - {M_{g2}}$	${M_{c1}} + c - {S_c} - {L_{c2}} - {N_c} - {L_{c1}} - {M_{c2}}$	${M_e} - {H_{e1}} - {N_e} - {B_e}$

Thus, it can be determined that 1 (0, 0, 0), 2 (0, 0, 1), 3 (0, 1, 1), 4 (0, 1, 0), 6 (1, 0, 1), and 7 (1, 1, 0) are not evolutionarily stable equilibrium points. The stability strategy for the two remaining points, 5 (1, 0, 0) and 8 (1, 1, 1), was analyzed as described hereinafter.

Scenario 1: the evolutionary stable equilibrium point 5 (1, 0, 0) is reached when ${M_{g1}} - {S_{g1}} - {L_{c1}} - {M_{g2}} - {S_{g2}}$ < 0, ${N_c} + {L_{c1}} - {M_{c1}} - c + {M_{c2}}$ < 0, and ${N_e} + {L_{c2}} + {B_e} - {M_e} + {H_{e2}}$ < 0. The “strict regulation” strategy was developed because when the government regulates with strictness, the sum of the increase in appreciation by older adults for the government, in government credibility and performance, and in fines for breach of trust of providers far outweighs the cost of strict regulation. Moreover, although the government provides financial subsidies for providers to carry out the smart senior care technology services, the “breach of trust” strategy becomes effective when the sum of the total profit and additional benefits gained by the breach of trust is much greater than the cost of operations and the fines submitted by providers to the government. The “no use” strategy becomes effective for older adults when the costs of using the services are higher than the incentives, subsidies, and advantages afforded by the government’s strict regulation.

Scenario 2: when ${N_c} + {M_{g1}} + {N_e} - {S_{g1}} - {M_{g2}}$ < 0,${M_{c1}} + c - {S_c} - {L_{c2}} - {N_c} - {L_{c1}} - {M_{c2}}$ < 0, and ${M_e} - {H_{e1}} - {N_e} - {B_e}$ < 0, the evolutionary stable equilibrium point 8 (1, 1, 1) is reached. As aforementioned, the government receives appreciation from older adults and an improvement in its credibility and performance when it regulates with strictness; thus, the “strict regulation” approach is adopted when the government’s gains outweigh the benefits that it delivers to providers and the costs associated with regulation. When the recognition and brand benefits that a provider receives for being trustworthy surpass the compensation and fines that must be paid to older adults and the government for a breach of trust, the “trustworthy” strategy is used. For older adults, both the government and technology service providers affect their strategic decisions: the government offers financial subsidies to older adults who choose to use smart senior care services, and when the government strictly regulates the service quality of providers, it rewards older adults who actively use smart senior care services. Meanwhile, the providers offer services that bring convenience, physical health, and spiritual satisfaction to older adults. Thus, when all these benefits of the services to older adults outweigh the purchase cost, the “use” strategy emerges.

### Simulation results

This study's parameter setting is partially based on actual senior care organization situations from Anhui Province, China, as well as local governmental regulations. We choose Anhui Jing'an Nursing Court Demonstration Base as an example in this instance [50, 51]. It has 301 beds and charges between CNY 2,679 to CNY 8,779 a month, with an average of CNY 5,000. We estimate that the range of bed occupancy is between 250 and 300 based on these facts. Furthermore, it costs 1.35 million annually to maintain trust for a smart senior care technology service provider to deliver high-quality service, and it costs CNY 750,000 annually to breach trust. The insurance liability range for the compensation limit in the event that the smart senior care technology service provider breaches trust is CNY 10000 to 200,000 [52]. Meanwhile, smart senior care demonstration bases that fulfill provincial requirements will be granted a minimum one-time award of CNY 200,000 [53]. Another part of the parameter setting is derived from relevant references. For instance, China's provincial-level civil affairs agencies offer older adults with good assessment abilities an annual operating subsidy of CNY 2,400 to 4,800 [54], and those who do not meet the standard will be fined between CNY 30,000 and 100,000 [55]. We also took into account the social benefits and costs of whole-process and nodal regulate by a Chinese municipal government, in addition to the additional benefits that resulted from the speculative actions of developers [56]. Ultimately, we determined the suitably defined parameters for this study by considering the advice of pertinent subject-matter experts as well as the previously provided data. The specific parameters are as follows: ${M_{c1}}$ = 135, ${M_{c2}}$ = 75, ${N_c}$ = 70, ${L_{c1}}$ = 10, $c$ = 100, ${L_{c2}}$ = 50, ${S_c}$ = 80, ${M_e}$ = 165,${H_{e1}}$ = 120, ${H_{e2}}$ = 60, ${B_e}$ = 30, ${N_e}$ = 70, ${M_{g1}}$ = 150, ${M_{g2}}$ = 100, ${S_{g1}}$ = 200, ${S_{g2}}$ = 75.

### Trends in the evolution of the government’s strategy

**Fig. 3:**
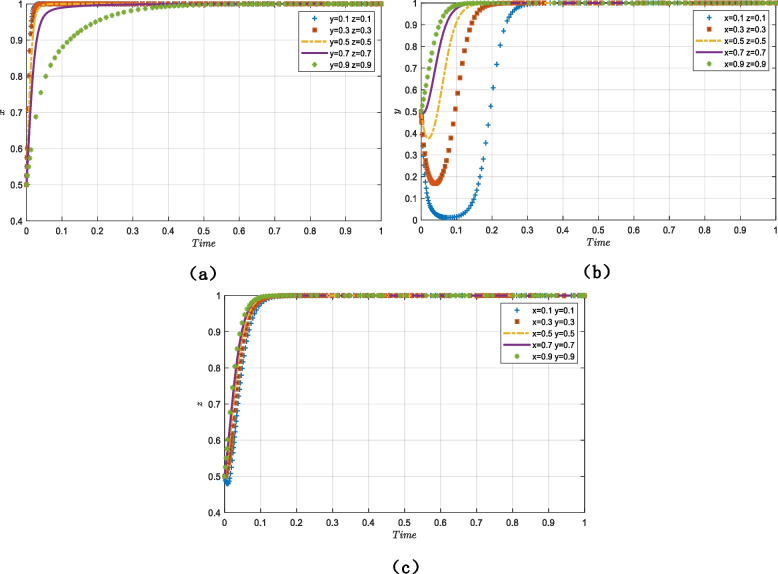
The impact of the initial willingness value on the evolutionary results (**a**) trends in the evolution of the government's strategy (**b**) trends in the evolution of the providers 's strategy (**c**) trends in the evolution of the older adults' strategy

As shown in Fig. 3a, to verify the evolutionary trend of $x$ over time, the initial willingness value of the government ($x$ = 0.5) is fixed at a constant and the values of $y$ and $z$ increase from 0.1 to 0.9. The government’s rate of convergence is impacted by the changes in the initial values for older adults and providers, but it eventually converges on 1 and adopts the “strict regulation” strategy. That is, the government’s rate of convergence will be slower and the evolutionary cycle will be longer when the initial values of older adults and smart senior care technology service providers are higher.

### Trends in the evolution of the providers’ strategy

As shown in Fig. 3b, to verify the evolutionary trend of $y$ over time, the initial willingness value of the providers ($y$ = 0.5) is fixed at a constant and the values of $x$ and $z$ increase from 0.1 to 0.9. When the government and older adults’ initial willingness values are between 0.1 and 0.5, the providers’ initial willingness value declines, then rises, and eventually stabilizes at 1; furthermore, as the initial values of $x$ and $z$ increase, the rate of development of $y$ accelerates and the evolutionary period shortens. Additionally, when the initial willingness values of the government and older adults were greater than 0.5, $y$ eventually stabilizes at 1. Therefore, although the main evolutionary line tendency of providers clearly changes, it has little impact on the outcomes.

### Trends in the evolution of older adults’ strategy

Figure 3c depicts the evolution trend of $z$ over time when the initial willingness value of older adults ($z$ = 0.5) is fixed and those of $x$ and $y$ increase from 0.1 to 0.9. Over time, the general trend of older adults’ strategy decision increases steadily with little variation in the rate of convergence and evolutionary period.

### Sensitivity analysis of model relevant parameters

#### Parameters influencing government strategy decisions

**Fig. 4:**
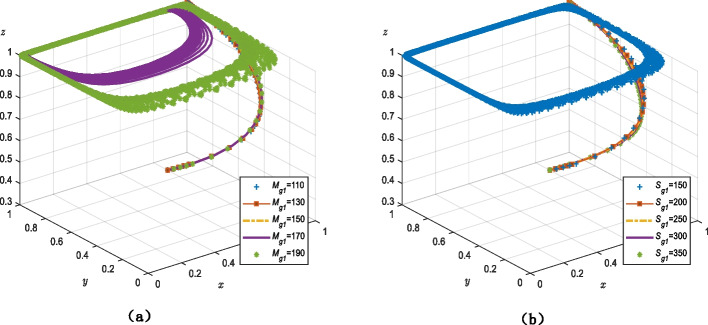
Parameters influencing the decision of government strategy (**a**) the cost of government strict regulation (**b**) the incremental gains in terms of its own performance, praise from older adults, and credibility gains when the government regulates strictly

Figure 4a demonstrates that when the value of ${M_{g1}}$ is between 110 and 130, the tripartite game system of smart senior care services always converges on (1, 1, 1). Additionally, a change in the value of ${M_{g1}}$ does not result in a change in the overall rate of the cycle of the system. There is also no system equilibrium point when the value of ${M_{g1}}$ is between 170 and 190, and the evolutionary stability strategy of the tripartite agents cannot be determined.

Figure 4b shows that when ${S_{g1}}$ is 150, the system is circular and there is no equilibrium point nor stability strategy for the tripartite game agents. When ${S_{g1}}$ is between 200 and 350, the system converges on (1, 1, 1) regardless of value. The evolutionary rate and duration are mostly stable and devoid of noticeable oscillations.

#### Parameters influencing providers’ strategy decisions

**Fig. 5:**
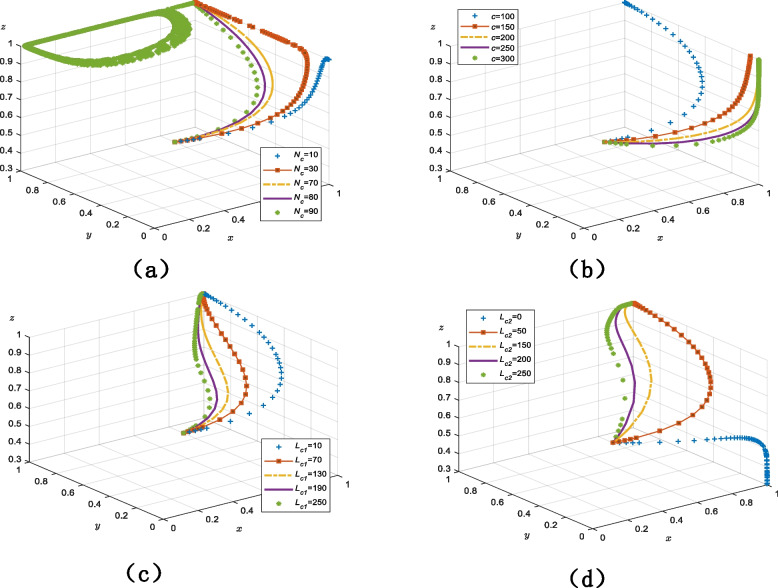
Parameters influencing the decision of strategy for providers (**a**) the government's reward for the “trustworthy” strategy of the provider when it is strictly regulated (**b**) additional benefits received by providers in the strategy of a breach of trust (**c**) the fines imposed by the government for “breach of trust” providers in the case of strict regulation (**d**) providers compensate older adults for breach of trust

As shown in Fig. 5a, the system converges on 0 when ${N_c}$ takes a value of 10 and the strategy decision of providers is “breach of trust.” The system converges on 1 when ${N_c}$ is in the range of 30–80 and providers adopt the “trustworthy” strategy. As governmental rewards increase, the system’s evolutionary rate increase rapidly. Additionally, when ${N_c}$ is 90, the system appears circular, and the tripartite game agents do not possess an evolutionary stability strategy.

Figure 5b demonstrates that when $c$ is 100, the equilibrium point of the tripartite game system is (1, 1, 1), and providers use the “trustworthy” strategy. When $c$ is between 150 and 300, the strategy shifts to “breach of trust.” Furthermore, system convergence occurs more quickly as the additional benefits increase, and if the additional benefits become very large, providers’ willingness to use the “breach of trust” strategy will increase.

Regardless of the value of ${L_{c1}}$, which ranges from 0 to 250, the system converges on 1—as shown in Fig. 5c—and providers adopt a “trustworthy” strategy. In addition, as ${L_{c1}}$ increases, the rate of evolution of the system increases.

Figure 5d demonstrates that older adults are not compensated for providers’ breach of trust when ${L_{c2}}$ = 0, and the older adults’ strategy is to “no use.” When ${L_{c2}}$ ranges between 50 and 250, the strategies of older adults and providers change and the system’s equilibrium point is (1, 1, 1). As the compensation for providers increases, the rate of evolution of the system increases.

#### Parameters influencing older adults’ strategy decisions

**Fig. 6:**
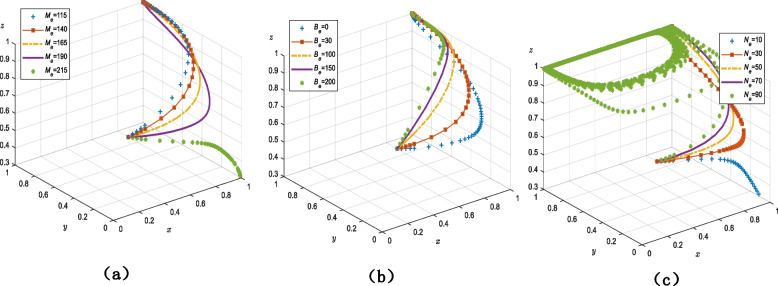
Parameters influencing the decision older adults strategy (**a**) the cost of older adults to purchase smart senior care services (**b**) the government's financial subsidy for older adults who use smart senior care services (**c**) strictly regulated government rewards older adults for actively using smart senior care services

As shown in Fig. 6b, the system converges on 1 and the evolution rate increases when ${M_e}$ fluctuates between 115 and 165. The system evolution rate slows down when ${M_e}$ is 190, but it still converges on (1, 1, 1) and the evolutionary outcome is unaltered. When ${M_e}$ is 215, the system’s equilibrium point is (1, 0, 0) and older adults engage in the “no use” strategy.

As shown in Fig. 6b, when ${B_e}$ ranges between 0 and 200, the system converges on 1. As government subsidies for older adults increase, so does the evolutionary rate at which older adults “use” the care services.

Figure 6c demonstrates that when ${N_e}$ is 10, the equilibrium point of the system is (1, 0, 0) and older adults engage in the “no use” strategy. However, when ${N_e}$ ranges from 30 to 70, older adults’ decision shifts to the “use” strategy. The system converges more quickly with the gradual increase in government rewards for older adults. The system turns circular when ${N_e}$ reaches 90, at which point there is no equilibrium point nor a stability strategy.

**Fig. 7:**
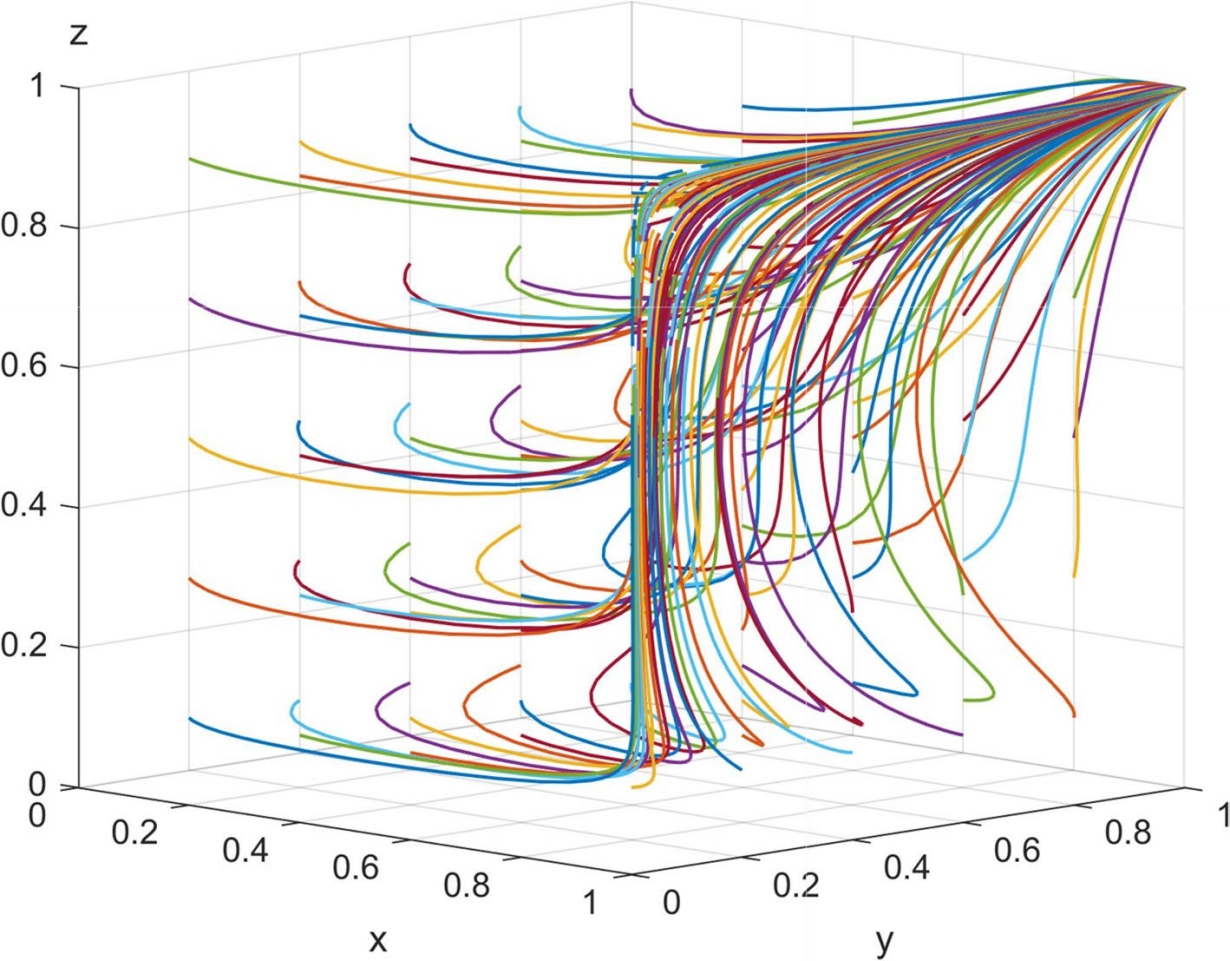
Plot of the results of 50 times of data evolution

Figure 7 demonstrates, based on data from Anhui Province and the reference assignment, that the equilibrium point of the tripartite game system of smart senior care services is obtained by evolving the array 50 times as (1, 1, 1), such that the main stability strategy is (strict regulation by the government, trustworthy providers, use by older adults). The results of the simulation analysis based on realistic situations are consistent with the discriminatory results obtained using Lyapunov’s First Method.

## Discussion

The simulation findings indicate that, first, each agent’s (i.e., government, smart senior care technology service providers, and older adults) strategy changes as the initial willingness values of the other two agents alter, albeit the system eventually converges on 1 regardless of the initial willingness value. Second, the synergistic mechanism of the tripartite governance agents in smart senior care is related to government incentives, penalties, and subsidies, smart senior care service cost, and additional benefits provided to service providers.

### The role of the government in a multi-agent governance system for smart senior care

First, from the perspective of the government, the overall evolutionary trends shown in Fig. 4a and b are similar; there were no discernible changes in the system’s evolutionary rate as the values rise and eventually form a cycle. This indicates that the government’s regulatory decisions are not highly impacted by the outcomes of strict regulation, namely changes in government monitoring costs and the advantages of this strategy (e.g., credibility and performance). This contradicts the results of other studies that contend that the probability of governmental regulation is inversely correlated with its expenses [57, 58] and positively correlated with reputational profits [59]. The inference is that the government, as the authority, continues to play a dominant role in the governance system through regulation regardless of interference from outside parameters, and that its role and functional attributes make it less susceptible to such interference. Therefore, the government can ultimately decide to regulate strictly regardless of changes in the strategies of the other agents, as depicted in Fig. 4a.

It is also important that the government provides only modest financial assistance because more assistance will simply increase financial burden and negatively impact social welfare. In this instance, the system is circular, as is demonstrated in Fig. 5a with ${N_c}$ as 90 and in Fig. 6c with ${N_e}$ as 90, making the agent’s strategy impossible to be determined. This conclusion is in line with other research' findings that excessive subsidies will result in a significant cost burden [60, 61]. As a result, the government ought to implement "dynamic subsidies" and "dynamic incentives" policies, as well as arbitrarily determine the proportion of incentives to subsidies.

In order to ensure the quality and safety of the services, the government should simultaneously continuously establish a sound regulatory system to standardize the management framework for senior care service providers and introduce social forces—such as the public, third-party regulators, etc.—to conduct assessment and testing. Additionally, the government should set up a support system that includes financial, technical, and training support to help smart senior care technology service providers improve their capabilities and provide the technical guidance services that older adults require. Finally, pertinent policies and regulations should be developed in order to set clear standards and direction for the development of smart senior care.

### The role of providers in a multi-agent governance system for smart senior care

Second, from the perspective of smart senior care technology service providers, providing them with extra benefits make them vulnerable to engaging in a “breach of trust” strategy [62]. This study implied that providers are a market force that chases profit maximization between by navigating public welfare and profit, making providers vulnerable to a “breach of trust” strategy. Therefore, in addition to offering high-quality services, smart senior care technology service providers should also intensify their marketing initiatives to encourage older adults to make purchases in order to strike a balance between costs and benefits and lessen providers' reliance on additional benefits. Meanwhile, an increase in the fines for a breach of trust, in the compensation for older adults [63], and in the offer of incentives [64] would relieve the financial pressure on providers and lead them to use a “trustworthy” strategy. It is important to note that the results of our study, which imposed fines for breach of trust, are different from those of our predecessors. Tsebelisg, Liu, and Zhang contend that punishing companies does not sufficiently constrain their violating behaviours to produce satisfactory outcomes [43, 65, 66]. Therefore, the government should make efforts and find methods to strengthen market regulations, market order regulations, service quality, improve the well-being of older adults using the services, and encourage providers to fulfill their social responsibilities.

Additionally, in order to provide more comprehensive and effective senior services, smart senior care technology service providers should keep innovating technologically and creating products and services suited to the needs of older adults. They should also cooperate and share with each other and increase publicity to encourage older adults to actively participate and provide feedback, as well as to continuously improve and optimize their offerings.

### The role of older adults in a multi-agent governance system for smart senior care

Finally, from the standpoint of older adults, it is possible to significantly increase their demand for and willingness to utilize smart senior care services by reducing service costs [67] and offering them financial subsidies and incentives [64]. Contrary to Tian's results, he argues that subsidies to producers are more beneficial than subsidies to users [68]. This may be because smart senior care services are relatively expensive for older adults or even beyond their financial capabilities, as these services involve large investments in technological development, equipment acquisition, maintenance, and expert personnel skills support. To reduce the burden on older adults, the government should provide them with more incentives and subsidies. This will increase their digital literacy and enable them to integrate better into modern society.

Older adults should actively engage in the usage and learning of smart senior care services, and comprehend and master pertinent technologies and knowledge. Simultaneously, they ought to offer input and recommendations to the government and service providers in order to help them enhance and optimize smart senior care services and encourage collaborative development. Additionally, they need defend their own legal rights and interests, strengthen the capacity of smart senior care services to identify, and stop fraud and information leaks.

### Limitations

The numerical settings for this study were sourced from Anhui Province, China, and the entire game system involves only the government, providers, and older adults. Thus, owing to the constrained data sources and agent parameter assumptions, the results of the simulation are not fully applicable at the national level. Future researchers should attempt to use other available methodologies and more agents to evaluate model results while considering the aforementioned limitations when utilizing simulations for assessment and prediction.

## Conclusion

This study analyzes and simulates the strategic decisions of the multi-agent collaborative governance system of smart senior care services in China using an evolutionary game model. The findings demonstrate that there are interactions between the strategy decisions of the government, smart senior care technology service providers, and older adults. Increasing penalties for defaulting providers, rewards for provider trustworthiness, and compensation for older adults can effectively restrain defaulting behaviors from providers. The willingness of older adults to use smart senior care services can be increased by reducing service costs, boosting government subsidies, and offering incentives.

These findings provide relevant data that can support the following: the regulation of the behavior of smart senior care technology service providers; an increase in older adults’ knowledge of smart senior care services; the clarification of the interests of multiple agents in the studied system; the improvement of governance effectiveness. They also provide ideas for resolving the complicated social issues that arise in multi-agent governance.

## Data Availability

All data generated or analyzed during this study are included in this published article.
